# Altered Intermittent Rhythmic Delta and Theta Activity in the Electroencephalographies of High Functioning Adult Patients with Autism Spectrum Disorder

**DOI:** 10.3389/fnhum.2017.00066

**Published:** 2017-02-20

**Authors:** Dominique Endres, Simon Maier, Bernd Feige, Nicole A. Posielski, Kathrin Nickel, Dieter Ebert, Andreas Riedel, Alexandra Philipsen, Evgeniy Perlov, Ludger Tebartz van Elst

**Affiliations:** ^1^Section for Experimental Neuropsychiatry, Department for Psychiatry and Psychotherapy, University Medical Center Freiburg, University of FreiburgFreiburg, Germany; ^2^Medical Campus University of Oldenburg, School of Medicine and Health Sciences, Psychiatry and Psychotherapy—University Hospital, Karl-Jaspers-KlinikBad Zwischenahn, Germany

**Keywords:** Asperger syndrome, autism spectrum disorder, EEG, adults, IRDA

## Abstract

**Background**: Autism spectrum disorder (ASD) is often associated with epilepsy. Previous studies have also shown increased rates of electroencephalographic (EEG) alteration in ASD patients without epilepsy. The aim of this study was to compare the rate of intermittent rhythmic delta and theta activity (IRDA/IRTA) events between high-functioning adult patients with ASD and matched healthy controls.

**Materials and Methods**: Routine EEG records of 19 ASD patients and 19 matched controls were screened for IRDA/IRTA using a fully data driven analysis with fixed thresholds. IRDA/IRTA rates before and after hyperventilation (HV) as well as the HV-induced difference in IRDA/IRTA rates (HV difference) were analyzed. For inter-group measures, we used the Wilcoxon rank sum test.

**Results**: Significantly increased HV difference was detected in the ASD group (*p* = 0.0497). However, the groups showed no difference in IRDA/IRTA rates before HV (*p* = 0.564) and after HV (*p* = 0.163).

**Conclusions**: The lack of any group differences regarding IRDA/IRTA before HV might be related to the fact that we only studied non-secondary high-functioning autism in a small sample of epilepsy-free adult patients. A significantly increased HV difference might be regarded as a marker of subtle neuronal network instability possibly causing short-term disturbances via local area network inhibition and long-term effects via epileptic encephalopathy.

## Background

High-functioning autism spectrum disorder (ASD) is a common neurodevelopmental and basic psychiatric disorder (Tebartz van Elst, 2013; Tebartz van Elst et al., 2013). Its prevalence varies between 1% and 2.7%, with increased rates in men (Brugha et al., 2011; Kim et al., 2011). ASD is characterized by long-lasting deficits in social communication, repetitive/stereotypical behavior, and special interests (Tebartz van Elst, 2013; Tebartz van Elst et al., 2013; Lai et al., 2014). Patients with high-functioning forms of ASD have normal or above-average intelligence quotients (IQs) and are able to use language on a superficial level (Tebartz van Elst, 2013; Tebartz van Elst et al., 2013). ASD is associated with high rates of psychiatric comorbidity. Most frequently, patients suffer from comorbid depression, anxiety disorder, obsessive-compulsive disorder and psychotic disorders. It is also closely linked to other neurodevelopmental diseases, such as attention deficit hyperactivity disorder (ADHD) and tic disorders (Lai et al., 2014). Moreover, ASD is associated with epilepsy (see next section; Lai et al., 2014). The clinical assessment for ASD involves personal and developmental history (interviews of parents or caregivers), behavioral observations, a physical examination and psychometric testing as well as optional neuropsychological tests, structured interviews, or observational measures. In many clinical centers, instrument-based diagnostics, including laboratory measurements, electroencephalography (EEG) and cerebral magnetic resonance imaging (cMRI) are also performed during clinical assessments for ASD (Tebartz van Elst, 2013). From a pathophysiological perspective, primary forms of ASD without a recognizable cause and secondary forms with a recognizable cause can be distinguished. Patients with primary forms often display a familial liability to autism however without a well-recognized genetic disorder or syndrome. In such cases, hereditary ASD is interpreted as a polygenetic phenomenon. Secondary forms may be triggered by mono- or oligogenetic disorders (e.g., fragile X syndrome, tuberous sclerosis, etc.) or syndromes (e.g., isodicentric chromosome 15, DiGeorge syndrome, etc.), or they may be acquired via brain disorders (e.g., encephalitis with epilepsy, traumatic brain injury; Tebartz van Elst, 2013; Tebartz van Elst et al., 2013). Specific psychotherapy programs were developed to improve social interaction among ASD patients (e.g., The Freiburg Asperger Specific Therapy Manual for Adult Patients or the FASTER program; Ebert et al., 2013). So far no specific psychotropic drugs are approved for treatment of the autistic core symptoms, but pharmacological treatment of comorbidities or specific symptoms may be useful. For example, patients with comorbid depression can be treated with antidepressants, and in patients with EEG alterations, aggressiveness and impulsivity psychotropic anticonvulsants such as carbamazepine may be useful; however, further research regarding treatment of ASD symptoms is needed (Tebartz van Elst, 2013; Tebartz van Elst et al., 2013; Hirota et al., 2014).

### Autism Spectrum Disorders and Epilepsy

The link between autism and epilepsy or EEG alterations is well known. In fact, several reviews explaining this connection were published in the last few years (Spence and Schneider, 2009; Berg and Plioplys, 2012; Ghacibeh and Fields, 2015; Lee et al., 2015). Epilepsy rates range from 5% to 46% in autistic patients (Spence and Schneider, 2009; Ghacibeh and Fields, 2015), and epilepsy was found to be associated with low IQ in many studies (e.g., Tuchman et al., 1991; Hara, 2007; Amiet et al., 2008; Spence and Schneider, 2009; Berg and Plioplys, 2012). Generalized tonic-clonic and complex partial seizures as well as absences were described (Spence and Schneider, 2009). In addition, one-third of childhood epilepsy cohorts fulfilled the psychometric criteria for ASD (Clarke et al., 2005). In ASD patients without epilepsy, increased rates of EEG pathologies ranging from 6.7% to 61% were reported (Ghacibeh and Fields, 2015). In groups of patients with ASD, epileptiform activity was detected in about 60% of children (Chez et al., 2006; Kim et al., 2006) and more rarely in adults (18% in Hara, 2007).

### Role of Intermittent Rhythmic Delta Activity (IRDA)

In this study, we investigated intermittent rhythmic delta and theta activity (IRDA/IRTA). IRDA was first described by Cobb (1945) and involves pathological EEG patterns with unclear significance (Brigo, 2011). Structural, metabolic, inflammatory, or epileptic reasons can cause theta activity (Cobb, 1945; Accolla et al., 2011; Brigo, 2011; van Vliet et al., 2012). Structurally, the delta rhythm might be generated in the thalamus or basal nuclei (Cobb, 1945). We earlier hypothesized that IRDA/IRTA can induce adaptive homeostatic processes, which could lead to functional alternations in neuronal networks (Tebartz van Elst et al., 2011, 2016a; Tebartz van Elst and Perlov, 2013; Endres et al., 2016).

### Rationale of Our Study

Given this theoretical background, the aim of our study was to compare IRDA/IRTA rates between primary ASD patients without epilepsy and matched controls. To create a homogeneous study cohort, only high-functioning patients with ASD were included. We hypothesized that we would find: (1) increased IRDA/IRTA rates in the resting EEGs of ASD patients; and (2) a significantly more pronounced increase in IRDA/IRDA after hyperventilation (HV) as a correlate for unstable cerebral networks in ASD patients.

## Participants and Methods

All patients and controls agreed to EEG examinations. EEG measurements were part of the routine clinical procedure for ASD patients. In the study, we included all available datasets from ASD patients of the Department of Psychiatry and Psychotherapy, University Medical Center Freiburg, between 2006 and 2012. The study was approved by the local ethics committee (Faculty of Medicine, Freiburg University, EK-Fr 233/14) and conformed to the principles of the Declaration of Helsinki. Control subjects were recruited from an earlier research project (Tebartz van Elst et al., 2014; van Elst et al., 2014; Endres et al., 2015; Maier et al., 2016).

### Patient Assessment

Only patients fulfilling the ICD-10 criteria for Asperger syndrome were included^1^. The diagnostic procedure was conducted by experienced senior consultant psychiatrists. All clinical information about comorbidity and somatic diseases was received from medical reports. We excluded all secondary ASD forms to create a homogeneous study sample. Psychometric measurements included the autism spectrum quotient (AQ; Baron-Cohen et al., 2001) and the empathy quotient (EQ; Baron-Cohen and Wheelwright, 2004) for autistic symptoms. In uncertain cases, the Autism Diagnostic Interview-Revised (Lord et al., 1994), the autism diagnostic observation schedule-generic (Lord et al., 2000), or behavioral observations were performed. For retrospective detection of ADHD symptoms in childhood, the Wender Utah Rating Scale (WURS-k) was used (Retz-Junginger et al., 2002).

### Control Group

Control subjects were recruited from an earlier project (Tebartz van Elst et al., 2014; van Elst et al., 2014; Endres et al., 2015; Maier et al., 2016). Therefore, the control group was well investigated using cMRIs; interviews to obtain patients’ demographics, psychosocial characteristics and medical histories; psychometrics (i.e., multiple-choice vocabulary intelligence test (MWT-B; Lehrl et al., 1995); WURS-k (Retz-Junginger et al., 2002), AQ (Baron-Cohen et al., 2001), and EQ scores (Baron-Cohen and Wheelwright, 2004); and semi-structured interviews (i.e., Mini International Neuropsychiatric Interview; Sheehan et al., 1998). Only controls without psychotropic medication, without neurological disorders, normal intelligence, no current axis I or II disorders, WURS-k scores of <30, and AQ scores of <30 were included.

### Matching Procedure

Between 2006 and 2012, we investigated 32 patients with ASD (20 males, 12 females). The control group included 34 healthy controls; one EEG was excluded due to technical reasons, one EEG was excluded due to incomplete psychometrics. To exclude age and gender effects, we automatically matched both variables. This resulted in 19 well-matched patient–control pairs (9 male pairs, 10 female pairs).

### EEG Acquisition

Topographical EEG was recorded using all 21 standard locations of the international 10–20 system (Klem et al., 1999). The recording reference was Fpz, ground Oz. Analog signals were recorded with a time constant of 0.3 s and a low pass of 70 Hz, sampled at 256 Hz and continuously stored for further processing. Off-line, the digital signals were filtered between 0.3 Hz and 45 Hz and downsampled to 100 Hz. EEG monitoring was performed for 11 min, and EEG recording was divided into three parts: first, a resting state was maintained for 6 min; second, a HV period was maintained for 3 min; and third, a post-HV period was maintained for 2 min. NEUROFILE software was used to register data^2^.

### EEG Analysis

The data analysis was performed using in-house software, avg_q^3^. The main steps were: (1) marking artifacts, including signal jumps and “blocking”, i.e., sections without signal variation in any channel; (2) independent component analysis (ICA) on the artifact-free data sections avoiding 5 s before and after any artifact marker (extended ICA; Lee et al., 1999; Makeig et al., 1999); (3) detection and correction of electrooculographic (EOG) artifacts by exclusion of EOG-related ICA components; and (4) detection of IRDA/IRTA. Empirically determined thresholds were used for the detection of phase and jump artifacts, optimizing detection and minimizing false positive findings. IRDA/IRTA was detected in any non-excluded independent component irrespective of topography. IRDA/IRTA detection was achieved by filtering the activation time series of all non-excluded independent components between 2 Hz and 7 Hz and thresholding for maximal amplitude between 25 μV and 245 μV. Only IRDA/IRTA candidates within the artifact-free EEG sections were considered. IRDA/IRTA rates were computed as events per minute separately for the intervals before and after HV.

### Statistical Analyses

For statistical analyses, we used Statistical Package for the Social Sciences, version 22 (SPSS 22^4^) and R, version 3.2.2^5^. The continuous variables of age, and psychometric scores were compared using two-sided independent sample *t*-tests. The gender ratio was calculated using Pearson’s two-sided chi-square test. IRDA/IRTA rates were corrected for age, using a linear model to estimate the predicted influence of subjects’ age on IRDA/IRTA rates. These estimates were then subtracted from the individual IRDA/IRTA rates. Group comparisons for the target variables (IRDA/IRTA before HV, IRDA/IRTA after HV, and difference in IRDA/IRTA from before HV to after HV [abbreviated as HV difference]) were performed using a Wilcoxon rank sum test (for inter-group comparisons). Moreover a parametric robust (non-parametric) repeated measures ANOVA (regular ranks) was performed using the npIntFactRep package in R to analyze the interaction between group and HV. We chose the robust repeated measure ANOVA since the assumption of homogeneity of variance was violated. In addition, medicated and unmedicated patients were compared using a Wilcoxon rank sum test. For outlier detection we performed a Tukey’s test. For correlation analyses of age corrected IRDA/IRTA rates with AQ und EQ scores, we calculated Spearman’s rank correlation. *P* < 0.05 served as the criterion of significance for all statistical analyses.

## Results

### Demographic and Psychometric Data

ASD patients and controls were matched in age and gender. In the patient group, 42% were medicated, mostly with antidepressants (37%). As expected psychometric autistic and ADHD scores were significantly increased in ASD patients (Table 1).

**Table 1:** **Demographic and psychometric information for the ASD and control groups**.

	ASD (*n* = 19) mean ± SD/*n*	Controls (*n* = 19) mean ± SD/*n*	Statistics
Age	38.37 ± 9.429	37.79 ± 8.331	*p* = 0.842
Gender	9 male: 10 female	9 male: 10 female	*p* = 1.0
Psychotropic drugs	None: 11 (58%)	None	
	Yes: 8 (42%)		
	- Antidepressants in 7/19 [37%]		
	- MPH in 2/19 [11%]		
	- Mood-stabilizer (lamotrigine): 1/19 [5%]		
	- Tranquillizer (zolpidem): 1/19 [5%]		
	- Atypical neuroleptics (promethazine): 1/19 [5%]		
AQ	38.00 ± 7.446	12.58 ± 4.959	*p* < 0.001
EQ	17.68 ± 9.363	44.63 ± 14.280	*p* < 0.001
WURS-k	26.42 ± 17.270	5.26 ± 5.536	*p* < 0.001

*Abbreviations: ASD, autism spectrum disorder; SD, standard deviation; IQ, intelligence quotient; M, male; F, female; MPH, methylphenidate; AQ, autism-spectrum quotient; EQ, empathy quotient; WURS-k, Wender Utah Rating Scale*.

### EEG Findings

No significant difference was found between ASD patients and control subjects regarding the rate of IRDA/IRTA before (*W* = 201, *r* = −0.175, *p* = 0.564) and after HV (*W* = 229, *r* = −0.283, *p* = 0.163). However, significantly increased HV difference was detected in the ASD group (*W* = 248, *r* = −0.364, *p* = 0.0497; Table 2). The comparison between medicated and unmedicated patients showed no differences in the frequency of IRDA/IRTA before (*W* = 42, *r* = −0.0277, *p* = 0.9039), after HV (*W* = 53, *r* = −0.1576, *p* = 0.492), and for HV difference (*W* = 52, *r* = −0.1389, *p* = 0.5448). In the robust ANOVA the interaction group by HV was significant (*F*_(1,36)_ = 6.50, *p* = 0.015). Outlier detection led to the exclusion of one ASD value for before, after and HV difference. The Wilcoxon test remained significant for the HV difference (*W* = 229, *r* = −0.3372, *p* = 0.0403) and not significant for IRDA/IRTA rates before (*W* = 182, *r* = −0.1455 *p* = 0.3763) and after HV (*W* = 210, *r* = −0.2538, *p* = 0.1226).

**Table 2:** **IRDA/IRTA per min in the ASD group and the control group**.

	ASD (*n* = 19) mean ± SD	Controls (*n* = 19) mean ± SD	Statistics Wilcoxon
IRDA/IRTA per min before HV	1.155 ± 1.452	0.793 ± 0.841	*W* = 201
			*r* = −0.175
			*p* = 0.564
IRDA/IRTA per min after HV	3.594 ± 5.528	1.038 ± 1.303	*W* = 229
			*r* = −0.283
			*p* = 0.163
Difference between IRDA/IRTA per min after HV and before HV	2.439 ± 4.987	0.245 ± 1.052	*W* = 248
			*r* = −0.364
			*p* **= 0.0497**

*Abbreviations: ASD, autism spectrum disorder; HV, hyperventilation; IRDA, intermittent rhythmic delta activity; IRTA, intermittent rhythmic theta activity. Significant *p*-values are written in bold*.

### Analysis of Dimensional Associations

The correlation analysis in the ASD group showed no significant correlations between IRDA/IRTA and autism scores (AQ, EQ).

## Discussion

The main finding of our study is the significantly increased HV difference in primary non-syndromal ASD patients. Contradictory to our hypothesis, we found no significant difference between the ASD and control groups in terms of the IRDA/IRTA rate in resting EEGs. Still the IRDA/IRTA-HV- difference might turn out to be a marker of discrete but possible relevant neuronal network instability.

### Comparison to Previous Studies

To our knowledge, this is the first study analyzing IRDA/IRTA in ASD patients. Our findings (significant increase in HV difference) are in line with earlier reports of increased rates of EEG alterations for children and adults with autism. In most previous studies, there was no differentiation between EEG alterations before and after HV. However, the absence of EEG alterations before HV is contrary to most findings (Spence and Schneider, 2009; Ghacibeh and Fields, 2015). The most likely explanation for this is the fact that we concentrated on high-functioning non-secondary autism in adults while most other previous studies did not differentiate between secondary and primary autism. Therefore other study groups contained also secondary and syndromal autism which is known to be more closely linked to epilepsy.

Furthermore most studies have focused on children, mostly with mental retardation (see overviews from Kawasaki et al., 1997; Spence and Schneider, 2009; Ghacibeh and Fields, 2015). Although some studies included adult patients with autism (e.g., Tuchman et al., 1991; Kawasaki et al., 1997; Giovanardi Rossi et al., 2000; Hughes and Melyn, 2005; Chez et al., 2006; Hartley-McAndrew and Weinstock, 2010), studies focusing only on adult cohorts are rare. Hara observed 130 patients with autistic disorder or atypical autism. In adulthood (18–35 years), 25% exhibited epileptic seizures; 68% of the epileptic group revealed epileptiform EEG findings before onset of epilepsy, 18% of the non-epileptic group exhibited epileptic discharges, and lower IQs were observed in the epileptic group (Hara, 2007). To our knowledge, no studies have analyzed high-functioning cohorts, as we did. EEG alterations in autism research might be influenced by different evaluation methods and the inclusion of different ASD subgroups (see “Discussion” Section about the nosology problem). For evaluation methods, we used routine EEGs. Earlier studies analyzing short routine EEGs found lower rates of EEG alterations (Hara, 2007). In contrast, high rates of EEG alterations were found with video EEG monitoring (epileptiform abnormalities in 59%; Kim et al., 2006) or 24 h digital overnight EEGs (epileptiform abnormalities in 61%; Chez et al., 2006).

### The Nosology Problem

If we look at previous EEG studies on autism, the nosology problem i.e., the fact that ASD comprises patient subgroups with diverse pathophysiologies, becomes apparent (Tebartz van Elst et al., 2006, 2013, 2014; Endres et al., 2015). Different subgroups of ASD can be distinguished by age, gender, course of disease, level of functioning, etiological variables and comorbidity. Regarding age, children, adolescent and adult patients must be distinguished; the dynamic changes in the brain during childhood and adolescence might result in different electrophysiological properties. In the current study, we investigated only adult patients and compared them with age-matched control subjects; therefore, developmental changes should be of no consequence. Regarding gender, earlier research reported increased prevalence of EEG alterations in young females on a meta-analytic-level (Amiet et al., 2008). In this study, we compared gender-matched ASD and control groups to exclude gender effects. Regarding the course of the disease, patients with early infantile autism, atypical autism, or Asperger syndrome must be distinguished. Developmental regression, which is observed in one-third of children with autism (Kawasaki et al., 1997), has been found to be associated with EEG alterations (Ghacibeh and Fields, 2015). EEG alterations were frequent in patients with regressive autism, and epileptiform activity was found more often in patients with regression than in patients without regression (Tuchman and Rapin, 1997; Lewine et al., 1999; Shinnar et al., 2001). However, other studies found no such differences in patients with developmental regression (Rossi et al., 1995; Chez et al., 2006; Giannotti et al., 2008). Regarding the level of functioning, high-functioning patients with normal or above-average IQs and effective use of language should be distinguished from low-functioning patients with low IQs and no communicative language. In support of this distinction a meta-analysis reported a higher prevalence of epilepsy in patients with intellectual disability (21.5%) compared to patients without intellectual disability (8%; Amiet et al., 2008). Regarding etiological variants, primary idiopathic forms of ASD must be distinguished from secondary non-idiopathic forms caused by acquired brain disorders, genetic disorders, or syndromal autism (Tebartz van Elst, 2013; Tebartz van Elst et al., 2013). Patients with secondary non-idiopathic forms were affected by epilepsy more often than patients suffering from primary idiopathic forms (Pavone et al., 2004; Miles et al., 2005). Finally, patients must be distinguished by comorbidity. Frequent developmental comorbidities are ADHD and tic disorders, and epilepsy itself is also an important neurological comorbid disorder (Lai et al., 2014). It can be assumed that EEG alterations are more prevalent in ASD patients with comorbid epilepsy (Hara, 2007). Until now, most studies have analyzed low-functioning children and adolescents (i.e., the typical patients of child and adolescent psychiatrists). To create a homogeneous study group and exclude developmental influences, we focused on high-functioning adults with primary variants and without relevant somatic comorbidities (particularly epilepsy but also a history of relevant birth complications or inflammatory brain disease). Such patients might suffer from a less severe form of ASD. In line with that idea, epileptiform activity was less common in patients with Asperger syndrome than in patients with more severe forms of autism (Mulligan and Trauner, 2014).

In summary, it can be noted that ASD is a heterogeneous disorder comprising many etiological subgroups and underlying pathophysiologies. The fact that we found no difference in the IRDA/IRTA rate before HV between groups might be explained by our inclusion of less severe forms of ASD without intellectual disability or developmental regression.

### Electrophysiological Perspective: The Cybernetic Role of Hyperventilation

In our study, the HV difference was significantly different. Therefore, the question of the role of HV arises. HV is an effective provocation method leading to decreased blood carbon dioxide levels, and therefore to cerebral vasoconstriction (Barker et al., 2012). This might result in EEG changes, such as physiological slowing, IRDA/IRTA, or epileptiform activity (Siddiqui et al., 2011; Barker et al., 2012). Interictal epileptiform activity was found particularly in patients with generalized epilepsy, but also in patients with focal epilepsy (Ahdab and Riachi, 2014; Kane et al., 2014). The role of EEG changes in patients without epileptic seizures is still unresolved. The phenomenon of HV-induced high-amplitude rhythmic slow activity with altered awareness in children without epilepsy was described as an epileptiform occurrence and physiological process (Barker et al., 2012). In this study, we focused on automatic detection of IRDA/IRTA, which is clearly pathological excitatory neuronal network activity (Tebartz van Elst et al., 2016a).

### Clinical View: The Effects of IRDA/IRTA and Network Inhibition

The most important clinical question is whether there is a causal relationship between EEG alterations and ASD symptoms. We hypothesize that local area network inhibition could cause short-term effects and epileptic encephalopathy could cause long-term effects. Alternatively, EEG alterations might simply be an epiphenomenon of a pathophysiological process responsible for ASD (Spence and Schneider, 2009).

#### Short-Term Effects: Local Area Network Inhibition

As described above, IRDA/IRTA is a pathological excitatory neuronal network activity. If such primary excitatory activity is focal and frequent enough, it could trigger a secondary homoeostatic reaction to which we have referred in previous publication using the term local area neuronal network inhibition (LANI). These putative inhibitory processes are conceptualized as a cybernetic homoeostatic mechanism of the brain to keep the functional equilibrium of the local neuronal network. While discrete and distributed LANI might not present with any clinical symptoms, ongoing suprathreshold LANI could in fact cause such symptoms. The nature of such phenomena then depends on the location of inhibited brain regions or anatomical loops (Tebartz van Elst et al., 2011; Tebartz van Elst and Perlov, 2013). Ongoing LANI in the frontal lobe might lead to stimulus satiation, and LANI in the left temporal lobe might trigger hallucinations.

#### Long-Term Effects: Epileptic Encephalopathy

Neuronal network instability as represented by IRDA/IRTA activity may lead to short term LANI effects illustrated above. But it may also cause long term effects stimulating the brain to restructure itself into a connectivistic pattern that minimizes the spread of epileptic activity (Tebartz van Elst et al., 2016b). Frequent epileptiform patterns can also interfere with normal neuronal physiology and brain development, disrupting various neurocognitive processes (i.e., plasticity, memory encoding and language processing) and causing developmental delays, regression or disruption. While this notion is controversial, respective results have been observed for other established syndromes (Holmes and Lenck-Santini, 2006; Ghacibeh and Fields, 2015). For instance, the Landau–Kleffner syndrome is characterized by language regression in normally developing children and is associated with EEG alterations mainly during sleep (Tebartz van Elst and Perlov, 2013). Cognitive functioning in patients with epileptic encephalopathy improves following normalization of their EEGs (Holmes and Lenck-Santini, 2006). Studies on children showed that antiepileptic treatment of interictal epileptiform activity could improve psychosocial function (Binnie, 2003; García-Peñas, 2011). On the background of such observations the question arises if the IRDA/IRTA-HV difference might serve as a biomarker indicating pathophysiological relevant neuronal network instability in a given patient.

### Limitations

#### Study Cohort

The study was retrospective; we included only patients from our clinic with ASD that were diagnosed between 2006 and 2012. To create a homogeneous study group, we included only adult patients with primary high-functioning forms of ASD. Patients with comorbid epilepsy and atypical autism were excluded from the study. In our previous studies on ASD, we established study cohorts with normal or above-average IQs as a filter for primary forms of ASD (Riedel et al., 2014; Tebartz van Elst et al., 2014). In the current study, IQ values were not available from all patients. To exclude secondary forms of ASD, the available clinical information was checked precisely. Additionally, we included medicated and unmedicated patients, which might have influenced our results. However, the IRDA/IRTA rates in medicated and unmedicated patients did not differ in statistical comparisons. Moreover, the total sample size could have been larger than 38, and further studies with increased numbers of subjects should be performed to confirm our findings. To subtract the possible variance of age and gender, we performed pairwise matching for these variables, which created highly comparable patient and control groups.

#### EEG Measurements and Statistical Analyses

**Figure 1:**
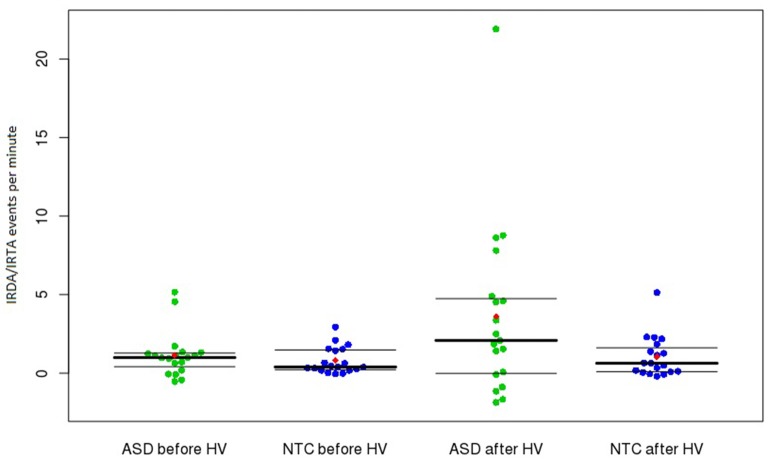
**Frequency of IRDA/IRTA events before and after HV in the ASD and control groups.** Presented are medians with upper and lower quantiles of corrected IRDA/IRDA rates. The use of age-corrected IRDA/IRTA-rates could result in negative corrected densities (i.e., IRDA/IRTA rates < 0). The red dots show the means. Abbreviations: ASD, autism spectrum disorder; HV, hyperventilation; IRDA, intermittent rhythmic delta activity; IRTA, intermittent rhythmic theta activity; NTC, neurotypical controls.

We assessed routine 11-min EEG studies. Repeated EEG recordings, sleep and sleep deprivation measurements, or video EEG telemetry probably would have led to higher rates of EEG alteration. Therefore, our findings should be regarded as representing the minimum detection rate in this sample. The data analysis was performed completely automatically and independent of an investigator. Thus we can exclude elements of rating bias. The method is published on the Internet and is therefore accessible to the reader. Regarding statistical analyses, there is potential to criticize our inclusion of outliers (Figure 1). However, we believe that these outliers could present the subgroup of subjects with network instability. In these patients (and in all the other patients), no secondary explanation (e.g., epilepsy or brain disease) of the IRDA/IRTA rates was found. The inclusion of these subjects in the statistical calculation resulted in a non-normal distribution of the IRDA/IRTA rates, which was considered in the statistics through the application of a Wilcoxon rank sum test. However, even with using outlier detection, the results remained significant for the HV difference.

## Conclusion

The primary finding of our study is a significantly increased HV difference in non-syndromal, high-functioning, epilepsy-free adult ASD patients. Further studies analyzing EEG alterations in larger cohorts of high-functioning adults with ASD are needed. An increased rate of IRDA/IRTA might be a neurophysiological marker of neuronal network instability which could have etiological and predictive relevance. For example it might modify cerebral information processing via short-term effects in terms of LANI or long-term effects via epileptic encephalopathy. The therapeutic implication of our findings is still unclear. Treatment with antiepileptic medication might be effective in such constellations. However, to prove this assumption, further clinical research with blinded, placebo-controlled intervention trials of ASD patients with EEG alterations are necessary.

## Author Contributions

LTvE, EP, DEn and SM initiated the study. BF developed the software for the EEG analysis. DEn, SM, NAP and BF conducted the data analysis. DEn and SM wrote the article. All authors were crucially involved in the theoretical discussion and performing of the manuscript. All authors read and approved the final version of the manuscript.

## Funding

Parts of the study were funded by the German Federal Ministry of Science and Education (ADHD-NET: 01GV0605, 01GV0606). The rest of the study was financed in-house by the Department of Psychiatry and Psychotherapy at the Medical Center of the University of Freiburg.

## Conflict of Interest Statement

AP Received advisory board fees from Lilly, advisory board, and lecture fees from Medice, Novartis, and Shire, congress support from Servier, and a travel grant from Lundbeck. She has also authored books and articles on adult ADHD published by Elsevier, Hogrefe, Schattauer, MWV, Kohlhammer, and Karger. LTvE Lectures, work shops or travel grants within the last 3 years Eli Lilly, Medice, Shire, UCB, Servier, and Cyberonics. The other authors declare that the research was conducted in the absence of any commercial or financial relationships that could be construed as a potential conflict of interest.

## Footnotes

^1^http://www.who.int/classifications/icd/en/GRNBOOK.pdf

^2^www.neurolite.ch/?q=en/products/eeg/coherence-software

^3^https://github.com/berndf/avg_q

^4^www-01.ibm.com/software/analytics/spss

^5^www.r-project.org

## Abbreviations

ADHD: attention deficit hyperactivity disorder
AQ: autism spectrum quotient
ASD: autism spectrum disorder
cMRI: cerebral magnetic resonance imaging
EEG: electroencephalography
EOG: electrooculography
EQ: empathy quotient
ICA: independent component analysis
IQ: intelligence quotient
IRDA: intermittent rhythmic delta activity
IRTA: intermittent rhythmic theta activity
HV: hyperventilation
MWT-B: multiple-choice vocabulary intelligence test
WURS: Wender Utah Rating Scale.
